# Datasets of 16S rRNA gene amplicon sequences, metabolites, and soluble immune components in bronchoalveolar lavage samples from severe asthmatic and age-matched control children

**DOI:** 10.1016/j.dib.2025.112359

**Published:** 2025-12-07

**Authors:** Mélanie Briard, Blanche Guillon, Eric Venot, Marta Grauso, Aurélia Bruneau, Marie-Noëlle Rossignol, François Fenaille, Florence Castelli, Muriel Thomas, Guillaume Lezmi, Maria Leite-de-Moraes, Karine Adel-Patient, Vinciane Saint-Criq

**Affiliations:** a Université Paris-Saclay, CEA, INRAE, UMR Département Médicaments et Technologies pour la Santé (DMTS)/SPI/Laboratoire d’Immuno-Allergie Alimentaire, 91191 Gif-sur-Yvette, France; b INRAE, Université Paris-Saclay, AgroParisTech, UMR1319 Micalis Institute, 78350 Jouy-en-Josas, France; c Université Paris Saclay, INRAE, AgroParisTech, UMR1313 GABI, 78350 Jouy en Josas, France; d Université Paris-Saclay, CEA, INRAE, UMR Département Médicaments et Technologies pour la Santé (DMTS)/SPI/Laboratoire innovations en spectrométrie de masse pour la santé, MetaboHUB, 91191 Gif-sur-Yvette, France; e AP-HP, Hôpital Necker-Enfants Malades, Service de Pneumologie et Allergologie Pédiatriques, Paris, France; f Université de Paris Cité, Institut Necker Enfants Malades, Equipe Immunorégulation et Immunopathologie, Inserm UMR1151, CNRS UMR8253, F-75015, Paris, France

**Keywords:** Microbiome, Metabolomics, Cytokines, Children, Bronchoalveolar lavage

## Abstract

Severe asthma (SA) is a heterogeneous condition characterized by multiple phenotypes, each characterized by different endotypes. Understanding the mechanisms occurring in the lungs of children with SA can help in understanding pathogenesis and in providing the most effective therapeutic strategies. This article describes microbiota, metabolites, and soluble immune components assessed in bronchoalveolar lavage (BAL) fluids from children with severe asthma (*n* = 20) and age-matched disease controls (*n* = 10). The article includes: (i) the protocol used to process BAL samples for 16S rRNA gene amplicon sequencing, metabolomic profiling and immune components assays; (ii) the bioinformatics steps applied to 16S rRNA and metabolomics dataset; (iii) an overview of the raw 16S rRNA gene amplicon sequencing data, presented as ASV and affiliation tables, raw data from untargeted metabolomics and the abundances of each of the eighty eight metabolites annotated with the highest confidence level, and concentrations of seventy three cytokines and of total IgG, IgA and IgE. Each dataset is available in the INRAE data repository (https://entrepot.recherche.data.gouv.fr/dataverse/inrae) with respective DOI: MICROBIOTA: 10.57745/LL3TFW, METABOLITES: 10.57745/1L8VRI, IMMUNE COMPONENTS: 10.57745/JOOGRQ

These datasets provide valuable resources for further investigating the molecular mechanisms underlying severe asthma in children and its trajectories. They also offer the potential to identify a local signature of severe asthma through complementary multi-omics analyses and to discover local biomarkers associated with asthma endotypes. Datasets can also be reused to compare with other cohorts (children or adults) or to serve as reference datasets for other pulmonary diseases.

Specifications Table

**Table:** 

Subject	Health Sciences, Medical Sciences & Pharmacology
Specific subject area	16S rRNA gene amplicon sequencing, metabolomics, and immune components analyses of bronchoalveolar lavage (BAL) samples obtained from a human French paediatric cohort presenting or not severe asthma
Type of data	16S rRNA gene amplicon sequencing: Raw (fastq files) and analysed (ASV table, taxonomy table, and sample metadata table) data Metabolomics: raw data from liquid chromatography coupled with high resolution mass spectrometry (LC—HRMS; .mzXML files) and LC—HRMS peak areas of 88 annotated metabolites and their characteristics (.xlsx files) Immune components: concentrations of 73 cytokines and of total IgG, IgA and IgE (.xlsx file)
Data collection	BALs were collected during endoscopy. Preparation of purified DNA libraries and sequencing of amplicons were carried out using MiSeq Illumina technology LC—HRMS was performed on an Ultimate 3000 chromatographic system coupled to a Q-Exactive mass spectrometer fitted with an electrospray source operating in the positive ionization mode. Cytokines and antibodies (IgG and IgA) were assayed using multiplexed commercial or in-house validated (IgE) immunoassays
Data source location	BALs were collected in Hospital “Necker Enfants Malades” in Paris (France); Fastq files were collected in Jouy-en-Josas (INRAE, France), metabolomics files and Cytokines/antibodies were collected in Saclay (CEA, MetaboHub, France)
Data accessibility	**16S rRNA amplicon sequencing** Repository name: data INRAE Data identification number: doi:10.57745/LL3TFW Direct URL to data: https://entrepot.recherche.data.gouv.fr/dataset.xhtml?persistentId=doi:10.57745/LL3TFW
	**Metabolomics data** Repository name: data INRAE Data identification number: 10.57745/1L8VRI Direct URL to data: https://doi.org/10.57745/1L8VRI **Soluble immune components data** Repository name: data INRAE Data identification number: 10.57745/JOOGRQ Direct URL to data: https://doi.org/10.57745/JOOGRQ
Related research article	K. Adel-Patient, M. Grauso, R. Abou-Taam, B. Guillon, C. Dietrich, F. Machavoine, M. Briard, N. Garcelon, H. Faour, A. Neuraz, C. Delacourt, T.J. Molina, M. Leite-de-Moraes, G. Lezmi, A Comprehensive Analysis of Immune Constituents in Blood and Bronchoalveolar Lavage Allows Identification of an Immune Signature of Severe Asthma in Children, Front. Immunol. 12 (2021). 10.3389/fimmu.2021.700521

## 1 Value of the Data

• These data provide information on lower airways microbiota, metabolome, and immunity in French children with severe asthma or in disease-control children
• BAL sampling in children is ethically and logistically challenging, making these datasets particularly valuable for paediatric respiratory research, especially for understanding severe, treatment-resistant asthma
• Researchers can use these datasets to compare our paediatric asthma airway profiles with those from other diseases or geographical areas, or from adult cohorts
• Our datasets may support the development of targeted therapies and translational models.
• The datasets facilitate investigation into how microbial communities and their metabolic products may influence inflammation, immune responses, and airway function in asthma.

## 2 Background

Severe asthma (SA) is defined by persistent airway inflammation, hyperresponsiveness to environmental triggers and uncontrolled exacerbations despite high-dose of inhaled corticosteroids, additional controllers, proper medication use and avoidance of environmental exposure like tobacco [1]. While SA patients present with similar clinical features, multiple underlying inflammatory mechanisms have been described. Thus, this multifaceted pathology is primarily classified as T2-high (eosinophilic) and T2-low (non-eosinophilic) endotypes, though recent studies reveal more complex immune mechanisms involving T1, T2, and T17 inflammation [[2], [3], [4]].

In addressing the inadequate response to conventional treatment in patients with SA, biologics have emerged as a new type of treatment in recent years. However, to implement such therapies effectively at the individual level, it is crucial to understand the disease’s phenotypes – the observable characteristics influenced by both genetics and the environment – and their underlying endotypes that involve distinct cellular and molecular mechanisms [5,6].

There is therefore a need for further characterization of the local lung environment to help understand the endotype-phenotype relationship and the pathogenesis of SA in the paediatric population.

## 3 Data Description

The data is divided into 3 datasets named “MICROBIOTA”, “METABOLITES”, and “IMMUNE COMPONENTS”, each containing raw and/or analyzed data folders. In all datasets, each patient received a code using “CL” for the name of the cohort (CLASSE), followed by a 2-digit number. Each number was attributed incrementally at recruitment. Each code is unique, and each unique code relates to the same patient across the 3 datasets. Codes range from CL01 to CL34 as samples from four recruited children (samples CL02, CL10, CL18, CL28) were not available or not exploitable.

The MICROBIOTA dataset is entitled “Dataset of 16S rRNA Gene Amplicon Sequences in Bronchoalveolar Lavage Samples from Severe Asthmatic and Age-Matched Control Children”. In this dataset, the CLASSE_RAW_DATA.zip file contains all 16S rRNA amplicon sequencing raw data as fastq files for 3 extraction blanks and 30 patient samples. Sample accession numbers for the 16S rRNA amplicon sequencing, including forward (R1) and reverse (R2) reads, range from BLC1_S103_L001_R1_001.fastq to CL34_S231_L001_R2_001.fastq. The analyzed sequencing data, including tables for ASV abundance and ASV taxonomic assignment (obtained from FROGS and DADA2 analyses), and sample metadata for use, are available in the CLASSE_ASV_TABLE_[FROGS/DADA2].xlsx, CLASSE_TAX_TABLE_[FROGS/DADA2].xlsx, and CLASSE_SAMPLE_TABLE.xlsx files, respectively.

The METABOLITES dataset is entitled “Dataset of untargeted metabolomics analysis of bronchoalveolar lavage samples from severe asthmatic and age-matched control children”. In this dataset, the file contains LC—HRMS raw data from 30 patient samples, from internal quality control and its dilutions, from buffer only and extraction blank (CLASSE_METABOLOME_RAW_DATA.zip). The Intensities_annotated_metabolites_BAL.xlsx file contains peak areas (i.e., integrated ion signal areas) of 88 annotated metabolites detected in each of the 30 patient samples. Indeed, metabolite levels are expressed as normalised peak areas suitable for comparative and multivariate analyses within a same acquisition batch: they are relative but not quantitative, and are unitless. The Metabolites_characteristics.xlsx file describes the acquisition characteristics (*m/z*, retention time, confidence of annotation), chemical class and KEGG identifier of each metabolite.

The IMMUNE COMPONENT dataset is entitled “Dataset of concentrations of soluble immune components in bronchoalveolar lavage samples from severe asthmatic and age-matched control children”. In this dataset, the immune_components_BAL.xlsx file contains concentrations of 73 cytokines, and total IgG, IgA and IgE assessed in each of the 30 patient samples.

## 4 Experimental Design, Materials and Methods

### 4.1 Experimental design

Twenty school-aged children with severe asthma (SA) and ten children with chronic respiratory disorders other than asthma and requiring endoscopy (age-matched disease-control subjects, called non-asthmatic, NA), were included. All patients were mainly from the Paris region in France. Clinical data, patient characteristics, and ethical statements are described in our previous studies [3,4,7]. Children with SA used higher doses of ICS (Inhaled corticosteroids) and had a higher post-bronchodilator Forced Expiratory Volume in 1 s (FEV1) over Forced Vital Capacity (FVC) ratio, and higher blood eosinophil counts than controls.

Broncho-alveolar lavages were collected during endoscopy, which was performed at least four weeks after an infection or asthma exacerbation. Lavages were recovered after injection of 3 mL/kg of body weight of sterile NaCl 0.9 %. The standard bronchoscopy procedure was followed for each patient.

Cytology, bacterial cultures, and immunofluorescence testing for common viruses were performed as part of the clinical assessments [4,8]. Among children with SA, bacterial cultures were positive in five samples (three for *Haemophilus influenzae*, one for *Streptococcus pyogenes*, and one for *Staphylococcus aureus*). In NA children, bacterial cultures were also positive in five samples (three for *Haemophilus influenzae*, one for *Haemophilus influenzae* and *Moraxella catarrhalis*, and one for *Staphylococcus aureus*). Viruses were found in four SA children (two with rhinovirus, one with both adenovirus and parainfluenza virus, and one with respiratory syncytial virus) and in one NA child (non-SARS-CoV-2 coronavirus).

**Fig. 1:**
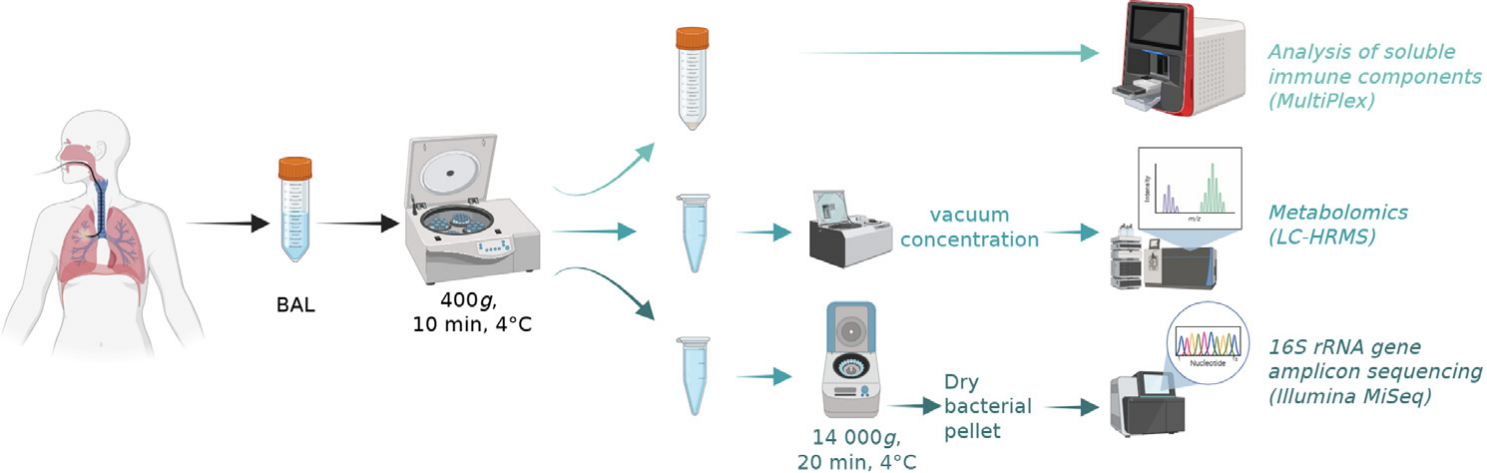
Bronchoalveolar lavage sample collection and processing strategy prior to the analyses of soluble immune components, metabolites, and 16S rRNA amplicon sequences. Created in BioRender.

Recovered lavages were kept on ice and transferred to the research laboratory within 3–4 h. After centrifugation (400×*g*, 10 min, 4 °C), bronchoalveolar lavage supernatants were collected (thereafter called BALs). Two BAL aliquots were stored at −80 °C for subsequent metabolomics and immune soluble components analyses. One of the aliquots was concentrated under vacuum before analysis by liquid chromatography coupled to high-resolution mass spectrometry (LC—HRMS). Remaining BALs were further centrifuged (14,000×g, 20 min, 4 °C), and dry pellets were stored at −80 °C until DNA extraction and 16S rRNA gene amplicon sequencing (Fig. 1).

### 4.2 16S rRNA gene amplicon sequencing

#### 4.2.1 DNA extraction from BAL pellet

All samples were processed in a safety level 2 lab, under a Class II Biosafety Cabinet. DNA was extracted in sterile conditions from dry pellets, using the QIAamp Power Fecal DNA kit and following the manufacturer’s recommendations (Qiagen; Courtaboeuf, France). Chemical then mechanical (FastPrep-24™, 2 × 40 s, 4 m/s) lysis was performed. After centrifugation (3 min, 18,000×g) and protein precipitation, the DNA was purified on spin filter columns. DNA was eluted with 2 × 50 µL of water. Three extraction blanks were prepared alongside biological samples to evaluate the contaminants from reagents, consumables, and the surrounding environment. DNA quantity was assessed using Qubit™ (Invitrogen, Villebon-sur-Yvette, France). The DNA concentration of the samples ranged between 0.164 and 174 ng/μL. All three extraction blanks showed DNA concentrations below the Qubit detection limit.

#### 4.2.2 PCR amplification of the V3-V4 hypervariable region of the 16S rRNA gene

For all samples and extraction blanks, the V3-V4 hyper-variable region of the 16S rRNA gene was amplified using the primers 341F (5′ CTT TCC CTA CAC GAC GCT CTT CCG ATC TCC TAC GGG NGG CWG CAG 3′) and 785R (5′ GGA GTT CAG ACG TGT GCT CTT CCG ATC TGA CTA CHV GGG TAT CTA ATC C 3′) [9]. Amplifications were carried out using the Mastermix 16S/18S Basic kit (Molzym GmbH): for each sample/control, DNA (4 µL) was mixed with 12 µL H_2_O, 10 µL Mastermix, 0.5 µL 341F primer, 0.5 µL 785R primer and 0.8 µL MolTaq polymerase. PCR was performed with an initial denaturation step at 94 °C for 1 min, followed by 40 cycles of denaturation at 94 °C for 1 min, annealing at 65 °C for 1 min, and extension at 72 °C for 1 min. The reaction concluded with a final extension step at 72 °C for 10 min to ensure complete amplification of the target DNA sequences. Amplifications were confirmed by visualisation of a 450 bp fragment on a 1 % agarose gel.

#### 4.2.3 16S rRNA gene amplicon sequencing

Preparation of purified DNA libraries and sequencing of amplicons were carried out on an Illumina MiSeq platform using a MiSeq Reagent Kit v3 (Illumina, USA) to generate 2 × 250 bp paired-end reads, at the @bridge sequencing platform (@BRIDGe platform, INRAE, Jouy-en-Josas, http://abridge.inra.fr/). Using a limited cycle PCR, Illumina sequencing adapters and index barcodes were added to the amplicon target. Sequencing was performed in two independent batches, including both internal controls and replicates of several samples.

#### 4.2.4 Bioinformatics analyses

Bioinformatics analyses were carried out using FROGS (Find Rapidly OTUs with Galaxy Solution) on the Galaxy Migale platform [10] or combining DADA2 v.1.36.0, and FROGS 4.1.0 (see below).

FROGS. A pre-process step (Galaxy Version 4.1.0+galaxy1) was performed to merge, denoise, and dereplicate sequences. The maximum read length for Read 1 and Read 2 were set to 250 bp, and the maximum rate of mismatches in the overlap region was set at 0.1. Paired-end reads were merged using VSEARCH and unmerged reads were excluded. Dereplicated sequences were then clustered using Clustering SWARM with an aggregation distance (d) of 1. Although FROGS traditionally performs OTU clustering, when using the SWARM algorithm with *d* = 1, the resulting clusters correspond to amplicon sequence variants (ASVs), as they represent unique denoised sequences differing by at most one nucleotide [11]. This thus produced an ASV-containing BIOM file and a corresponding FASTA file.

DADA2. A second analysis was performed using R v.4.5.1, combining DADA2 v.1.36.0, and FROGS 4.1.0. Adapters were first removed using cutadapt v. 5.0. Reads were then filtered using the DADA2 filterAndTrim function, applying a truncation length of 230 bp for 16S forward and reverse reads. The error model was estimated using the learnErrors function and sequence inference was performed with the DADA2 core sample inference. Forward and reverse reads were then merged with a minimum overlap of 20 bp. The resulting sequences were assembled into a sequence table using makeSequenceTable, generating both BIOM and FASTA files.

Each BIOM+FASTA file pair was then processed as follows: chimeras were detected and removed following FROGS v4.1.0 guidelines using VSEARCH v2.17.0. Clusters/ASVs with a prevalence <80 or a global abundance below 0.0005 % were removed from subsequent analyses using FROGS filters. Potential contaminants were identified using the PhiX databank. The remaining clusters/ASVs were taxonomically assigned with BLAST against the SILVA Pintail100 16S rRNA database (version 138.1) [12]. Manual affiliation curation was performed using the Affiliation Explorer application in Shiny Migale (https://shiny.migale.inrae.fr/app/affiliationexplorer) when multiple taxa were associated with the same Cluster/ASV. The datasets from the two independent sequencing batches were then merged, and PCoA and PERMANOVA analyses were conducted to confirm that no significant differences existed between batches.

### 4.3 Metabolomic profiling by liquid chromatography coupled to high-resolution mass spectrometry (LC—HRMS)

#### 4.3.1 Sample preparation

In order to perform sample normalisation, total protein concentration (Pierce™ BCA Protein Assay Kit, Thermo Fisher Scientific) in BALs was first assessed. Protein concentrations ranged between 0.051 to 1.011 mg/mL. Then, 500 µL of BALs were concentrated (SpeedVac™) and reconstituted in ultrapure water to obtain a final concentration of 0.139 mg of total protein /mL. Proteins were then precipitated by adding 4 vol of MeOH for 1 vol of samples [13]. After 90 min of incubation on ice and centrifugation (20,000*×*g, 15 min, 4 °C), the resulting supernatants were collected and aliquoted. Quality control (QC) was prepared by pooling equal volumes of all samples. Finally, all the samples and QC were dried under nitrogen (RotorVap LV-Biotage, 30 °C, 5 bars).

#### 4.3.2 LC—HRMS analysis

Dried samples were resuspended in 108 µL of a solution consisting of 95 % water and 5 % acetonitrile, both containing 0.1 % formic acid. QC and diluted QC samples (1/2, 1/4 and 1/8) were prepared in the same solution. QC, diluted QC and 100 µL of each biological sample were spiked with 5 µL of an external standard mixture (Table 1), and 10 µL of the resulting samples were injected into the LC—HRMS system. LC—HRMS analysis was performed on an Ultimate 3000 chromatographic system coupled to a Q-Exactive (Orbitrap) mass spectrometer (Thermo Fisher Scientific, Courtaboeuf, France) fitted with an electrospray (ESI) source operating in the positive (ESI+) ionization mode. Ultra-high performance LC (UHPLC) separation was performed at 30 °C using a C18 column (Hypersil GOLD C18, 1.9 µm, 2.1 mm × 150 mm column, Thermo Fisher Scientific). Mobile phases were 100 % ultrapure water (phase A) and 100 % acetonitrile (phase B), both containing 0.1 % formic acid. Chromatographic elution was performed at a flow rate of 500 μL/min, starting with an isocratic step of 2 min at 5 % phase B, followed by a linear gradient from 5 to 100 % of phase B for the next 11 min. These proportions were kept constant for 12.5 min before returning to 5 % B over 4.5 min. The column effluent was directly introduced into the electrospray source of the mass spectrometer. Source parameters were as follows: droplet evaporation temperature, 280 °C; capillary voltage, 5 kV; sheath gas pressure and the auxiliary gas pressure set, respectively, at 60 and 10 arbitrary units, with nitrogen gas. The mass spectrometer operated in the positive ionisation mode at a mass resolution of 50,000 full width at half maximum (FWHM) at *m/z* 200with a detection from *m/z* 85 to 1000.

**Table 1:** List of external standards used for LC—HRMS analysis (with C18 column).

Compound	Elemental formula	Accurate Mass	Retention time (min)
^13^C_1_-Alanine	^13^C_1_^12^C_2_ H_7_ N O_2_	90.05103	0.88
Metformin	C_4_ H_11_ N_5_	129.10145	0.83
^15^N_1_-Aspartate	C_4_ H_7_^15^N_1_ O_4_	134.03454	0.81
^13^C_1_-Glucose	^13^C_1_^12^C_5_ H_12_ O_6_	181.06674	0.85
2-Aminoanthracene	C_14_ H_11_ N	193.08915	8.05
Amiloride	C_6_ H_8_ Cl N_7_ O	229.04789	3.1
Imipramine	C_19_ H_24_ N_2_	280.19395	7.5
Atropine	C_17_ H_23_ N O_3_	289.16779	5.2
Prednisone	C_21_ H_26_ O_5_	358.17802	6.75 and 7.00
Colchicine	C_22_ H_25_ N O_6_	399.16819	6.44
Dihydrostreptomycin	C_21_ H_41_ N_7_ O_12_	583.28132	0.72
Roxithromycin	C_29_ H_54_ O_10_ N_2_	590.37784	5.87 and 6.08

Diluted QCs were analysed in triplicate at the beginning of the sequence, while non-diluted QCs were introduced every six randomised biological samples for data normalisation and standardisation purposes. Buffer only (“Blanc”; injected 5 times at the very beginning and once at the end of the data acquisition) and an extraction blank consisting of sterile NaCl 0.9 % extracted and treated as samples, were also analysed for data filtration purpose.

#### 4.3.3 Data treatment and annotation

Raw data (.raw files) were manually inspected using the Qual-browser module of Xcalibur (version 4.1, Thermo Fisher Scientific) and then converted to .mzXML format using MSconvert (“ProteoWizard”, version 3.0.21079). Data extraction was performed using the XCMS software package deployed on the open-source web-based W4M platform (https://workflow4metabolomics.org/) (CLASSE_METABOLOME_RAW_DATA.zip) [14]. Filtration of the variables, as well as evaluation of quality metrics, were carried out using the phenomis R package (version 1.0.2) [15] . Features generated from XCMS were filtered according to the following criteria: (i) correlation between QC dilution factors and areas of chromatographic peaks (> 0.7), (ii) coefficient of variation of chromatographic peak areas of QC samples (< 30 %), and (iii) ratio of chromatographic peak areas of biological to that of blank samples (> 3). Chromatographic peak areas of each variable were corrected for signal drift by fitting a locally quadratic (loess) regression model to the QC values. Initially, 25,698 variables were extracted with the XCMS package, and 2972 variables were retained after filtering. Feature annotation was then performed along the 2972 variables using an in-house spectral database based on chemical standards analysed under the same analytical conditions [13,16]. To be annotated, features had to match accurately measured masses (± 10 ppm) and chromatographic retention times (± 45 s). Metabolite annotations were then confirmed by MS/MS analyses by matching MS/MS spectra to those included in our in-house spectral database, allowing the highest level of confidence (level 1 according to Metabolomic Standards Initiative [17]).

### 4.4 Immune mediators profiling

We quantified 73 cytokines and total IgG, IgA and IgE in BALs for each individual. Cytokines were analyzed using xMAP® Luminex technology and the associated apparatus (Bioplex®200, Biorad, Marnes-la-Coquette, France). chemokines (Bio-Plex Pro™ Human chemokine assays, 40-plex; BioRad) and 37 inflammation markers (Bio-Plex Pro™ Human Inflammation Panel 1, 37-plex; BioRad) were analysed in all BAL samples following the manufacturer’s recommendations. Samples were incubated with the beads for 18 h at +4 °C to increase sensitivity. IL-5 and IL-13 were not detectable or below the limit of quantification in most samples. Due to small redundancy between the kits, 73 immune soluble constituents were finally quantified for each sample: APRIL/TNFSF13, BAFF/TNFSF13B, sCD30/TNFRSF8, sCD163, Chitinase 3-like 1, CCL21 (6Ckine), CXCL13 (BCA-1), CCL27 (CTACK), CXCL25 (ENA-78), CCL11 (Eotaxin), CCL24 (Eotaxin-2), CCL26 (Eotaxin-3), CX3CL1 (Fractalkine), CXCL6 (GCP-2), GM-CSF, CXCL1 (Gro-α), CXCL2 (Gro-β), CCL1 (I-309), gp130/sIL-6Rβ, sIL-6Rα, IFNα2, IFNβ, IFNγ, IL-1β, IL-2, IL-4, IL-6, IL-8 (CXCL8), IL-10, IL-11, IL-12p40, IL-12p70, IL-16, IL-19, IL-20, IL-22, IL-26, IL-27 (p28), IL-28A (IFN-2λ), IL-29 (IFN-λ1), IL-32, IL-34, IL-35, CXCL10 (IP-10), CXCL11 (I-TAC), CCL2 (MCP-1), CCL8 (MCP-2), CCL7 (MCP-3), CCL13 (MCP4), CCL22 (MDC), MIF, CXCL9 (MIG), CCL3 (MIP-1α), CCL15 (MIP-1δ), CCL20 (MIP-3α), CCL19 (MIP-3β), CCL23 (MPIF-1), CXCL16 (SCYB16), CXCL12 (SDF-1α+β), CCL17 (TARC), CCL25 (TECK), LIGHT/TNFSF14, MMP-1, MMP-2, MMP-3, Osteocalcin, Osteopontin (OPN), Pentraxin-3, sTNF-R1, sTNF-R2, TLSP, TNFα, and TWEAK/TNFSF12. Total IgG and IgA were also analysed using the Bio-Plex Pro™ Human isotyping panel (BioRad) and total IgE using in-house specific immunoassays [18].

## Limitations

Limitations of the presented datasets include the lack of a positive control (i.e., known community standard) in the 16S rRNA gene amplicon sequencing data and relatively small statistical power. Moreover, the metabolites were annotated using an internal database containing mainly endogenous human metabolites, and the data were produced using a C18 column with detection in the positive ionisation mode only. The use of a complementary analytical LC—HRMS platform (e.g. metabolite separation on an HILIC column combined with detection in the negative ion mode) would likely bring additional relevant information.

## Ethics Statement

This study, involving human participants, was reviewed and approved by the “Comité de Protection des Personnes Ile de France 2” and written informed consent to participate in this study was provided by the participants’ legal guardian/next of kin.

## CRediT Author Statement

**Mélanie Briard**: Investigation, Data Curation, Writing - Original Draft. **Blanche Guillon**: Investigation, Data curation. **Eric Venot**: Data Curation. **Marta Grauso**: Investigation. **Aurélia Bruneau**: Investigation. **Marie-Noëlle Rossignol**: Investigation. **François Fenaille**: Investigation. **Florence Castelli**: Investigation. **Muriel Thomas**: Resources, Funding acquisition. **Guillaume Lezmi**: Resources, Funding acquisition. **Maria Leite-de-Moraes**: Resources, Funding acquisition. **Karine Adel-Patient**: Supervision, Investigation, Data Curation, Writing - Original Draft, Funding acquisition. **Vinciane Saint-Criq**: Supervision, Investigation, Data Curation, Writing - Original Draft

## Data Availability

https://entrepot.recherche.data.gouv.fr/dataverse/inraeDataset of 16S rRNA Gene Amplicon Sequences in Bronchoalveolar Lavage Samples from Severe Asthmatic and Age-Matched Control Children (Original data). https://entrepot.recherche.data.gouv.fr/dataverse/inraeDataset of untargeted metabolomics analysis of bronchoalveolar lavage samples from severe asthmatic and age-matched control children (Original data). https://entrepot.recherche.data.gouv.fr/dataverse/inraeDataset of concentrations of soluble immune components in bronchoalveolar lavage samples from severe asthmatic and age-matched control children (Original data).
